# Chiral Flavanones from *Amygdalus lycioides* Spach: Structural Elucidation and Identification of TNFalpha Inhibitors by Bioactivity-guided Fractionation

**DOI:** 10.3390/molecules17021665

**Published:** 2012-02-08

**Authors:** Raffaella Gaggeri, Daniela Rossi, Michael S. Christodoulou, Daniele Passarella, Flavio Leoni, Ornella Azzolina, Simona Collina

**Affiliations:** 1 Department of Drug Sciences, University of Pavia, Viale Taramelli 12, Pavia 27100, Italy; Email: raffaella.gaggeri@unipv.it (R.G.); daniela.rossi@unipv.it (D.R.); ornella.azzolina@unipv.it (O.A.); 2 Department of Organic and Industrial Chemistry, University of Milan, via Venezian 21, Milano 20133, Italy; Email: m.christodoulou@unimi.it (M.S.C.); daniele.passarella@unimi.it (D.P.); 3 Italfarmaco Research Center,Viale dei Lavoratori 54, Cinisello Balsamo (MI) 20092, Italy; Email: F.LEONI@italfarmaco.com; 4 Center for Studies and Researches in Ethnopharmacy (C.I.St.R.E.), University of Pavia, via Taramelli 12, Pavia 27100, Italy

**Keywords:** *Amygdalus lycioides* Spach, bioassay-guided fractionation, chiral flavanones, structural elucidation, phytochemical fingerprint, TNFα blockers

## Abstract

Phytochemical investigation on the *Amygdalus lycioides* Spach branchelets resulted in the isolation of four chiral flavanones: (*2R*,*3R*)*-*Taxifolin, (*2R*,*3R*)**-**aromadendrin, (*S*)-5,7,3',5'-tetrahydroxyflavanone and (*S*)*-*naringenin. The flavanones were isolated by semi-preparative HPLC, their structures elucidated based on spectroscopic data and their absolute configuration assigned. As a part of our ethnobotanical-directed search for novel TNFα inhibitors, the bioassay-guided fractionation of the *n*-hexane-acetone (*n*-Hex-Ac, 1:1 v/v) *Amygdalus lycioides* Spach branchelets extract was performed. In this way, (*S*)-naringenin was identified as the constituent responsible for the TNFα blocking effect, being effective *in vitro* and *in vivo* after oral administration. This is the first investigation on bioactive secondary metabolites of *Amygdalus lycioides* Spach branchelets.

## 1. Introduction

*Amygdalus lycioides* Spach [Family: Rosaceae, Genus: Amygdalus, Subgenus: Dodecandra, Species: lycioides; Syn.: Prunus lycioides C.K. Schneid] is an endemic species extending into South Anatolia [1]. In Iranian folk medicine it is commonly known as “Badam Talkh kuhi” and has been used as anti-inflammatory and antimicrobial remedy since ancient times [2]. In spite of the widespread use of *Amygdalus lycioides*, no investigations on its bioactive secondary metabolites or on its phytochemical fingerprint were carried out so far. As a part of our ethnobotanical-directed search for novel drugs, *Amygdalus lycioides* was recently studied by performing a bioassay guided extraction [3]. Particularly, during the early phase of biological screening, the extract obtained from *Amygdalus lycioides* dried branchelets using *n*-hexane-acetone (*n*–Hex-Ac, 1:1 v/v) as extraction mixture and applying a microwave assisted solvent extraction (MASE) procedure (henceforth called the MASE extract), gave rise to the best results in terms of antimicrobial, free-radical scavenging and anti-inflammatory activities. Of particular interest was the anti-inflammatory activity, exerted via a tumor necrosis factor alpha (TNFα)-blocking mechanism [3]. To date TNFα blockers represent a major advance in the treatment of chronic inflammatory diseases, such as rheumatoid arthritis, bowel diseases and psoriasis [4]. The most important TNFα blockers currentlly used in the clinical practice are biotechnological molecules, such as monoclonal antibody (infliximab) and receptor fusion protein (etanercept). They appear to be relatively safe during the short-term treatment. Currently, there are not enough data to determine whether patients treated with these TNFα blockers have a greater risk of lymphoma or skin cancer [5]. Accordingly, the search of small molecules acting as TNFα inhibitors with favorable safety profile represents one of the major goals for scientists operating in the field of inflammation diseases treatment [6,7]. Based on these considerations and our previous results, herein we report a deeper investigation of the MASE extract of *Amygdalus lycioides* branchelets in terms of: (1) isolation and structural elucidation of the main secondary metabolites and (2) bioassay-guided fractionation of the extract in order to identify the constituents responsible for the anti-TNFα effect.

## 2. Results and Discussion

**Figure 1:**
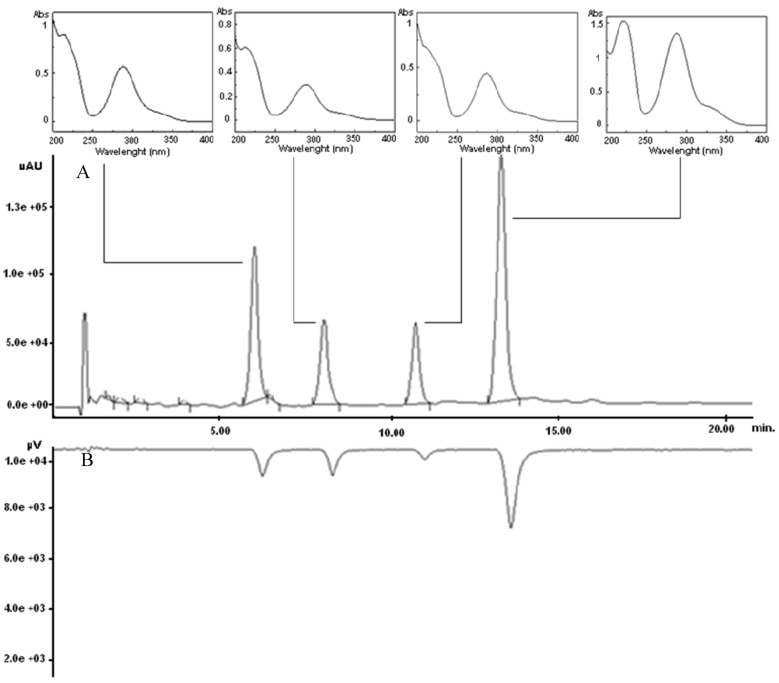
HPLC-UV/PAD/CD chromatogram (290 nm) of *Amygdalus lycioides* extract. **A**: UV trace (with UV spectra of each principal peak); **B**: CD trace.

The air-dried branches of *Amygdalus lycioides* Spach, defatted with petroleum ether [8], were extracted by using n-Hex-Ac (1:1 v/v) in a multimode microwave apparatus, according to our already developed methodology [3]. The crude extract was then subjected to analytical characterization by HPLC analysis. Taking into account our previous experiences [9,10], a rapid chromatographic method was developed by using a high performance liquid chromatograph with a ultraviolet photodiode array detector (HPLC-UV/PAD) coupled on-line with a Circular Dichroism (CD) detector, which is a powerful tool for a rapid detection of chiral compounds naturally occurring in crude extracts. Among the tested columns (Supelcosil LC-18, μBondapak C-18, Metasil C-18, Kromasil C-18, Chromolit Speed-ROD RP-18) the Chromolit Speed-ROD RP-18, made from a single piece of high-purity polymeric silica gel, gave rise to the best results in terms of both peak resolution and analysis time. The optimized HPLC method allowed us to draw the analytical fingerprint of the MASE extract, which revealed four noticeable peaks with retention time ranging from 6 to 13 min, corresponding to the four principal phytocomponents (compounds **1–4**) of the extract (Figure 1A). The nearly identical UV spectra profiles of **1–4** suggested that they may belong to the same phytochemical class. In details, they showed a maximum at 280–290 nm and a shoulder at 320 nm (Figure 1A), corresponding to the π→π* and n→ π* acetophenone chromophore transitions, characteristics of flavonoid skeleton [11]. Additionally, the online coupling of HPLC/CD yielded directly the CD signal of the resolved peaks, thus providing useful information on their chiroptical properties. Interestingly, in the HPLC-CD chromatogram **1–4** appeared as negative peaks (Figure 1B), suggesting that all of them were present in the MASE extract in enantiomeric form.

After analytical characterization, the MASE extract underwent inspection for: (1) structural identification of the main phytocomponents and (2) bioassay-guided fractionation, both at the same time.

**Figure 2:**
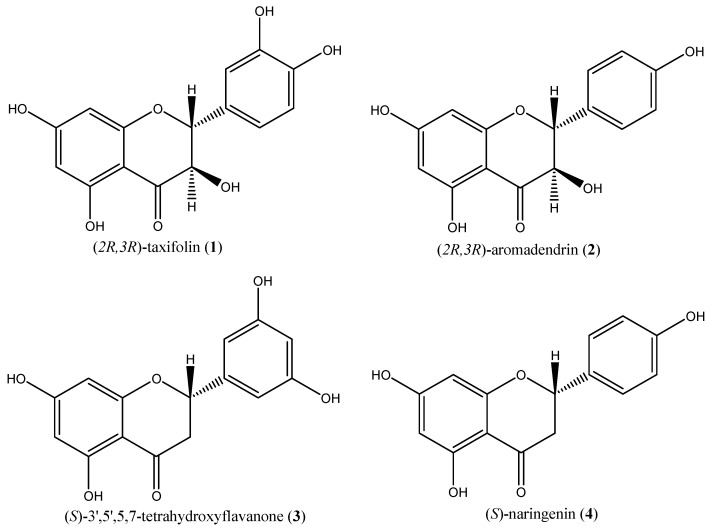
Structures of compounds **1–4**.

To isolate phytocomponents, a direct purification of the crude extract *via* semi-preparative HPLC was performed. To this end, the experimental conditions of the analytical method were appropriately scaled-up to semi-preparative scale using Chromolit Speed-PREP RP-18. In this way, compounds **1**, **2**, **3** and **4** were recovered with a purity higher than 98% and in amounts sufficient for structural characterization (12–40 mg). The structures of compounds **1–4** were elucidated as (*2R*,*3R*)-taxifolin [12,13], (*2R*,*3R*)-aromadendrin [14,15], (*S*)-5,7,3',5'-tetrahydroxyflavanone [16,17,18] and (*S*)-naringenin [19,20], respectively (Figure 2), by comparison of the measured [α]_D_, 1D and 2D NMR, and HRESIMS data of these compounds with values in the literature. Although these compounds have been already found in different vegetal matrix, they were isolated from *Amygdalus lycioides* Spach branchelets for the first time.

**Figure 3:**
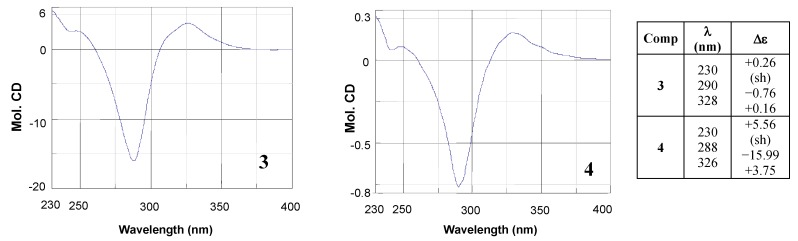
CD spectra and Δε data of **3** and **4**.

Concerning the absolute configuration assignment, for compounds **1**, **2** and **4** it was made by comparing ^1^H-NMR and polarimetric results with those reported in the literature. As regards compound **3**, the configurational assignment was performed by comparing the CD spectra of compounds **3** ([α]^20^_D_ = −12.0°, *c* = 0.2, MeOH) and **4** [(*S*)*-*naringenin], which is characterized by comparable substituents on stereogenic centre and a high structural similarity. As clearly seen in Figure 3, comparable Cotton effects were detected for compounds **3** and **4** in the range of wavelength 230–400 nm; indeed, in this region the CD curves showed the same pattern. Particularly, the CD curves of both **3** and (*S*)-naringenin exhibited a positive Cotton effect at the n**→**π* absorption band (λ = 328 nm) and a negative Cotton effect at the π**→**π* absorption band (λ = 290 nm), in accordance with CD data reported by Slade *et al.* [11] for flavanones with the (*S*) configuration at the C2. Accordingly, the (*S*) absolute configuration was proposed for **3**.

Since one of the main strategies used to speed up the identification of new lead compounds from vegetal matrix consists in the so-called bioactivity-guided fractionation, we decided to adopt this approach to identify the components of the MASE extract responsible for its TNFα-blocking activity. In this view, the crude extract was partitioned with solvents of different polarity, yielding four fractions: *n*-hexane, chloroform, ethyl acetate and *n*-butanol, respectively.

**Figure 4:**
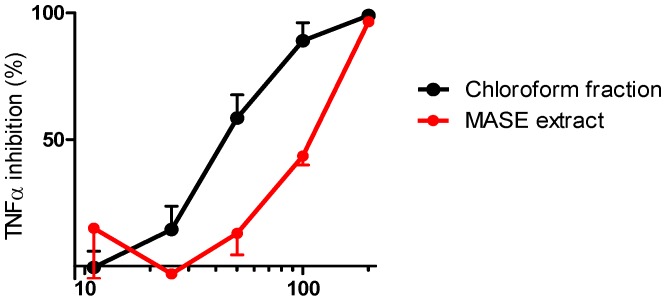
TNFα inhibitory activity of the MASE extract and the chloroform fraction.

The TNFα-blocking effect of each fraction was then evaluated in our *in vitro* assay, using human peripheral blood mononuclear cells (hPBMC) [3,21]. As reported in Figure 4, TNFα inhibitory activity was associated with the MASE extract and chloroform fraction (IC_50_ values of 109 and 55 μg/mL, respectively), while the *n*-hexane, *n*-butanol and ethyl acetate fractions did not exhibit any measurable inhibitory activity (data not shown). To confirm that the inhibitory effect was specific and to exclude that it was due to cytotoxicity, the vitality of the cells was also evaluated. Results evidenced that none of the analyzed samples showed significant cytotoxicity up to the dose of 200 μg/mL (cytotoxicity lower than 20%). 

**Figure 5:**
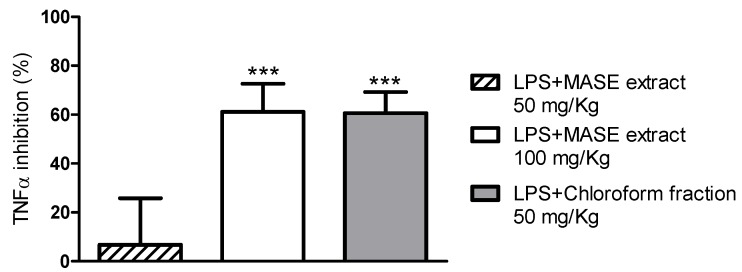
*In vivo* TNFα inhibitory activity of the MASE extract and the chloroform fraction; *** *p* < 0.0001 compared with LPS-treated mice.

Encouraged by the *in vitro* biological results, the anti-TNFα activity of the MASE extract and the chloroform fraction was also evaluated *in vivo* in a murine model of endotoxemia [22]. Both samples were administered orally to animals and their effect compared to that observed in vehicle-treated animals. Results are reported in Figure 5.

LPS-treated mice showed, as expected, a significant (*p* < 0.0001) increase of serum TNFα (9.8 ng/mL) that was undetectable in vehicle-treated animals (<31 pg/mL). The MASE extract, at 100 mg/kg, inhibited the TNFα production by about 60% (*p* < 0.0001), whereas at 50 mg/kg it was almost ineffective (the inhibition was about 7% and not significant). Interestingly, the chloroform fraction, administered at 50 mg/kg, inhibited serum TNFα similarly to the MASE extract administered at 100 mg/kg. Results obtained in our *in vivo* model of systemic inflammation are of great interest, firstly because they confirmed the *in vitro* data and, secondly, because they clearly proved the effectiveness of the chloroform fraction after oral administration. Overall, biological results strongly suggested that the chloroform fraction was enriched in the phytocomponents of the MASE extract responsible for the TNFα blocking activity.

Interestingly, the HPLC-UV analysis of the isolated fractions revealed that (i) (*S*)*-*naringenin was the constituent of the chloroform fraction, in which it was present with a chemical purity higher than 96% and an enantiomeric excess of 91.6% (the e.e. was determined according to Giorgio *et al*., [19]); (ii) (*2R*,*3R*)-taxifolin was the main constituent of the ethyl acetate fraction (chemical purity ≅60%), in which it was present together with (*2R*,*3R*)-aromadendrin (18%), (*S*)-5,7,3',5'-tetrahydroxy-flavanone (12%) and (*S*)-naringenin (10%); (iii) in the butanol fraction (*2R*,*3R*)-taxifolin, (*2R*,*3R*)-aromadendrin and (*S*)-5,7,3',5'-tetrahydroxyflavanone were present only in traces; (iiii) no UV-visible components were detected in the *n*-hexane fraction. Thus, considering both biological and analytical results, we can state that the phytocomponent responsible for the observed TNFα inhibitory activity is (*S*)*-*naringenin. Therefore, bioactivity-guided fractionation was successful in leading the identification of the compound responsible for the activity.

## 3. Experimental

### 3.1. Chemicals and Instrumentations

All solvents were from Carlo Erba (Milano, Italy). Acetonitrile was HPLC grade; the formic acid was of analytical grade. Extractions were carried out a multimode Microwave apparatus using a closed-vessel system (MARSX press, CEM Corporation, Matthews, NC, USA). Chromatographic analyses were performed by a Jasco system (Jasco Europe S.r.l., Cremella, LC, Italy) equipped with a Jasco AS-2055 plus autosampler, a PU-2089 plus pump and a MD-2010 plus multiwavelength detector coupled with a CD-2095 plus circular dichroism detector. Optical rotations were determined on DIP 1000 photoelectric polarimeter from Jasco (JASCO Europe, Cremella, LC, Italy) and recorded at room temperature using a 1dm cell and a sodium lamp. The NMR (^1^H-NMR, ^13^C-NMR, ^1^H-^1^H-COSY and ^1^H-^13^C HSQC) analyses were run in CD_3_OD-*d_4_*, unless otherwise stated, and were recorded on a Bruker 400 MHz spectrometer. Chemical shifts are reported in parts per million (δ) downfield from tetramethylsilane (TMS) as internal standard. EI mass spectra were recorded at an ionizing voltage of 6Kev on a VG 70-70 EQ. ESI mass spectra were recorded on FT-ICR APEX^II^ (Bruker Daltonics Billerica, MA, USA).

### 3.2. Plant Material

*Amygdalus lycioides* Spach branches were collected near Teheran (Iran) in September 2007 at an altitude of 2,400 m. A voucher specimen was deposited in the Herbarium of the University of Pavia (PAV 2007 11/15/01), Dipartimento di Scienze della Terra e dell'Ambiente, University of Pavia, Via S. Epifanio, 14-27100 Pavia, Italy. The air-dried plant material was stored in dark conditions, into air tight polyethylene container. 

### 3.3. Extraction and Fractionation

The air-dried branches (150 g) of *Amygdalus lycioides* Spach were pre-treated with petroleum ether 10% (w/v) (Okolo *et al*., [8]) and then extracted by using *n*-Hex-Ac (1:1 v/v) in a multimode microwave apparatus at 120 °C for 20 min, with a power of 800 W, according to our already developed methodology [3]. The extract was separated by filtration and solvent was evaporated to dryness under vacuum to 35 °C, yielding a dried residue (5.6 g). A portion (3.5 g) of the crude extract was suspended in distilled water and partitioned with *n*-hexane (200 mL × 3), chloroform (200 mL × 3), ethyl acetate (200 mL × 3) and *n*-butanol (200 mL × 3), yielding, after the evaporation of solvents, the corresponding fractions (250, 790, 1170 and 530 mg respectively).

### 3.4. Chromatography

#### 3.4.1. Analytical HPLC-UV/PAD/CD

Analytical chromatographic analyses were carried out using a Chromolith SpeedROD RP-18 endcapped column (50 mm × 4.6 mm, ID 3 mm, macropore size 2 μm, mesopore size 13 nm, Merck KGaA, Darmstadt, Germany) and a security guard H5-10C column. The mobile phase consisted of water containing 0.1% (v/v) formic acid (A) and acetonitrile (B); gradient elution (from 90% of A to 60% of A within 20 min, followed by a re-equilibration step of 5 min) was employed. The flow rate was set at 1 mL/min and detection was fixed at 290 nm for both UV and CD detectors. Each sample was dissolved in methanol (conc. = 3 mg/mL) and filtered with 0.45 μm GHP membrane before injection into the HPLC-system.

#### 3.4.2. Semipreparative HPLC

Semipreparative HPLC separations were performed on a Chromolith SemiPREP RP-18e (100 mm × 10 mm) column. The mobile phase was the same one used for the analytical separations. The flow rate was 9 mL/min and detection was fixed at 290 nm. Dried extract (7.5 mg) dissolved in acetonitrile was processed in a single run by collecting the four principal peaks. The process was repeated 20 times, yielding, after the evaporation of solvents, 31, 15, 12, 40 mg of peaks **1**, **2**, **3** and **4**, respectively. Each sample was filtered with 0.45 μm GHP membrane before injection into the HPLC-system.

*(2R*,*3R)-Taxifolin* (**1**): ^1^H-NMR, ^13^C-NMR, ^1^H-^1^H-COSY and ^1^H-^13^C HSQC see Supplementary info. EI-MS *m/z*: 304 [M]. Positive-ion HR-ESI-MS *m/z*: 327.0473 [M+Na]^+^ (calcd. for C_15_H_12_O_7_Na: 327.0475). [α]^20^_D_ + 12.68° (c = 0.2, MeOH). T.r. 6.5 (λ = 290). 

*(2R*,*3R)-Aromadendrin* (**2**): ^1^H-NMR, see Supplementary info. EI-MS *m/z*: 288 [M]. HR-EI-MS *m/z*: 288.0641 [M] (calcd. for C_15_H_12_O_6_: 288.0634). [α]^20^_D_ = +34.00° (c = 0.05, MeOH). T.r. 8.5 (λ = 290).

*(S)-5*,*7*,*3'*,*5'-Tetrahydroxyflavanone* (**3**): ^1^H-NMR, ^13^C-NMR, ^1^H-^1^H-COSY and ^1^H-^13^C HSQC see Supplementary info. EI-MS *m/z* 288 [M]. HR-EI-MS *m/z*: 288.0642 [M] (calcd. for C_15_H_12_O_6_: 288.0634). [α]^20^_D_ = −12.00° (c = 0.2, MeOH). T.r. 10.7 (λ = 290).

*(S)-Naringenin* (**4**): ^1^H-NMR, ^13^C-NMR, ^1^H-^1^H-COSY and ^1^H-^13^C HSQC see Supplementary info. Negative-ion HR-ESI-MS *m/z*: 271.0610 [M−H]^−^ (calcd. for C_15_H_11_O_5_: 271.0612). [α]^20^_D_ = −20.63° (c = 0.28, EtOH). T.r. 12.6 (λ = 290).

### 3.5. Biological Assays

#### 3.5.1. *In Vitro* anti-TNFα Assay

TNFα inhibition from human peripheral blood mononuclear cells (hPBMC) was carried-out as previously described by us (Gaggeri *et al.*, [3]; Leoni *et al*., [21]). Briefly, TNFα production was induced *in vitro* treating the cells with *E. coli* lipopolysaccharides (LPS) for 24 h in the presence of serial doses (1−200 μg/mL) of each sample (isolated fractions and extract) or medium (control cells). The TNFα levels in the cell supernatant was then measured by enzyme-linked immunosorbent assays (ELISA, m-TNFα DuoSet, R&D Systems, Minneapolis, MN, USA) and the inhibitory activity expressed as inhibitory concentration 50% (IC_50_). Statistical analysis by ANOVA was carried out using GraphPad Prism 5.0 Software. 

#### 3.5.2. *In Vivo* anti-TNFα Assay

The *in vivo* anti-TNFα activity was evaluated in a murine model of endotoxemia, as previously described (Leoni *et al*., [21]). Briefly, female CD1 mice, 20–22 g (Charles River Laboratories Italia S.r.l., Calco, LC, Italy), were randomly divided in groups of five animals and treated with the MASE extract (50 and 100 mg/kg) and the chloroform fraction (50 mg/kg). Both samples were dissolved in vehicle (saline plus 0.5% Tween 80) and administered by gavage. Control animals were treated with vehicle and 60 min later with 30 mg/kg of endotoxin (LPS *E. coli* O55B5, Sigma Aldrich S.r.l., Milano, Italy) intraperitoneally. Blood from each animal was drawn 90 min after endotoxin treatment and serum separated and frozen until TNFα evaluation by commercial ELISA assay (m-TNFα DuoSet, R&D Systems). Statistical analysis (*t*-test) was carried out using GraphPad Prism 5.0 Software. 

#### 3.5.3. Cytotoxicity Assay

The viability of cultured cells was assayed by using commercial Alamar Blue assay (Invitrogen, Milan, Italy) following producer indications. The dye was added after 18 h of incubation of the cells with the samples and fluorescence measured after 4 h by using a plate fluorometer (Victor Wallac Multilabel Counter 1420, Perkin-Elmer Italia S.p.A, Monza, Italy). Cytotoxicity was expressed as effective dose 50% (ED_50_). 

## 4. Conclusions

In summary, in the present communication we have reported for the first time the isolation and structural elucidation of the main phytocomponents of *Amygdalus lycioides* Spach branchelets and the identification of (*S*)*-*naringenin as TNFα inhibitor. Indeed, the dose dependent activity of (*S*)*-*naringenin exerted *in vitro* at non-cytotoxic concentrations as well as its *in vivo* efficacy suggest that this small molecule could represent a useful pharmacological tool in inflammatory conditions associated with increased TNFα production. Whether this activity is specific for TNFα or may be also extended to other pro-inflammatory cytokines, such as IL-1β and IL-6, should be investigated with *ad hoc* new studies. Although our preliminary results indicate that (*S*)*-*naringenin is orally active in our *in vivo* model of systemic inflammation, additional experiments are needed to evaluate its dose-response as well as its pharmacokinetic profile. Moreover, results on the pharmacological profile of (*2R*,*3R*)*-*taxifolin, (*2R*,*3R*)*-*aromadendrin and (*S*)-5,7,3',5'-tetrahydroxyflavanone will be reported in due course.

## Supplementary Materials

Supplementary materials can be accessed at: http://www.mdpi.com/1420-3049/17/2/1665/s1.
