# Supplementary material for: Chiral Flavanones from Amygdalus lycioides Spach: Structural Elucidation and Identification of TNFalpha Inhibitors by Bioactivity-guided Fractionation
Source: Molecules. 2012 Feb 8;17(2):1665–74. doi: 10.3390/molecules17021665 (PMC6268923; doi:10.3390/molecules17021665)

**Supplementary**

Chiral Flavanones from *Amygdalus lycioides* Spach: Structural Elucidation and Identification of TNFalpha Inhibitors by Bioactivity-guided Fractionation

Raffaella Gaggeri ^1,†^, Daniela Rossi ^1,†^, Michael S. Christodoulou ^2^, Daniele Passarella ^2^,
Flavio Leoni ^3^, Ornella Azzolina ^1^ and Simona Collina ^1,4^,*

^1^ Department of Drug Sciences, University of Pavia, Viale Taramelli 12, Pavia 27100, Italy;
E-Mails: raffaella.gaggeri@unipv.it (R.G.); daniela.rossi@unipv.it (D.R.); ornella.azzolina@unipv.it (O.A.)

^2^ Department of Organic and Industrial Chemistry, University of Milan, via Venezian 21, Milano 20133, Italy; E-Mails: m.christodoulou@unimi.it (M.S.C.); daniele.passarella@unimi.it (D.P.)

^3^ Italfarmaco Research Center,Viale dei Lavoratori 54, Cinisello Balsamo (MI) 20092, Italy;
E-Mail: F.LEONI@italfarmaco.com

^4^ Center for Studies and Researches in Ethnopharmacy (C.I.St.R.E.), University of Pavia, via Taramelli 12, Pavia 27100, Italy

**^†^** These authors contributed equally to this work.

***** Author to whom correspondence should be addressed; E-Mail: simona.collina@unipv.it;
Tel: +390-382-987-379; Fax: +390-382-422-975.

**List of contents**

1. ^1^H-NMR spectrum (CD_3_OD-*d_4_*, 400 MHz) of (*2R*,*3R*)*-*(+)**-**taxifolin (**1**)

2. ^13^C-NMR spectrum (CD_3_OD-*d_4_*, 400 MHz) of (*2R,3R*)*-*(+)**-**taxifolin (**1**)

3. ^1^H-^1^H-COSY spectrum (CD_3_OD-*d_4_*, 400 MHz) of (*2R*,*3R*)*-*(+)**-**taxifolin (**1**)

4. ^1^H-^13^C HSQC spectrum (CD_3_OD-*d_4_*, 400 MHz) of (*2R*,*3R*)*-*(+)**-**taxifolin (**1**)

5. ^1^H-NMR spectrum (CD_3_OD-*d_4_*, 400 MHz) of (*2R*,*3R*)**-**(+)*-*aromadendrin (**2**)

6.^1^H-NMR spectrum (CD_3_OD-*d_4_*, 400 MHz) of (*S*)-(−)-5,7,3',5'-tetrahydroxyflavanone (**3**)

7. ^13^C-NMR (CD_3_OD-*d_4_*, 400 MHz) of (*S*)-(−)-5,7,3',5'-tetrahydroxyflavanone (**3**)

8. ^1^H-^1^H-COSY (CD_3_OD-*d_4_*, 400 MHz) of (*S*)-(−)-5,7,3',5'-tetrahydroxyflavanone (**3**)

9. ^1^H-^13^C HSQC (CD_3_OD-*d_4_*, 400 MHz) of (*S*)-(−)-5,7,3',5'-tetrahydroxyflavanone (**3**)

10.^1^H-NMR spectrum (DMSO-*d_6_*, 400 MHz) of (*S*)*-*(−)**-**naringenin (**4**)

11. ^13^C-NMR spectrum (DMSO-*d_6_*, 400 MHz) of (*S*)*-*(−)**-**naringenin (**4**)

12. ^1^H-^1^H-COSY spectrum (DMSO-*d_6_*, 400 MHz) of (*S*)*-*(−)**-**naringenin (**4**)

13. ^1^H-^13^C HSQC spectrum (DMSO-*d_6_*, 400 MHz) of (*S*)*-*(−)**-**naringenin (**4**)

1. ^1^H-NMR spectrum (CD_3_OD-*d_4_*, 400 MHz) of (*2R*,*3R*)*-*(+)**-**taxifolin (**1**)


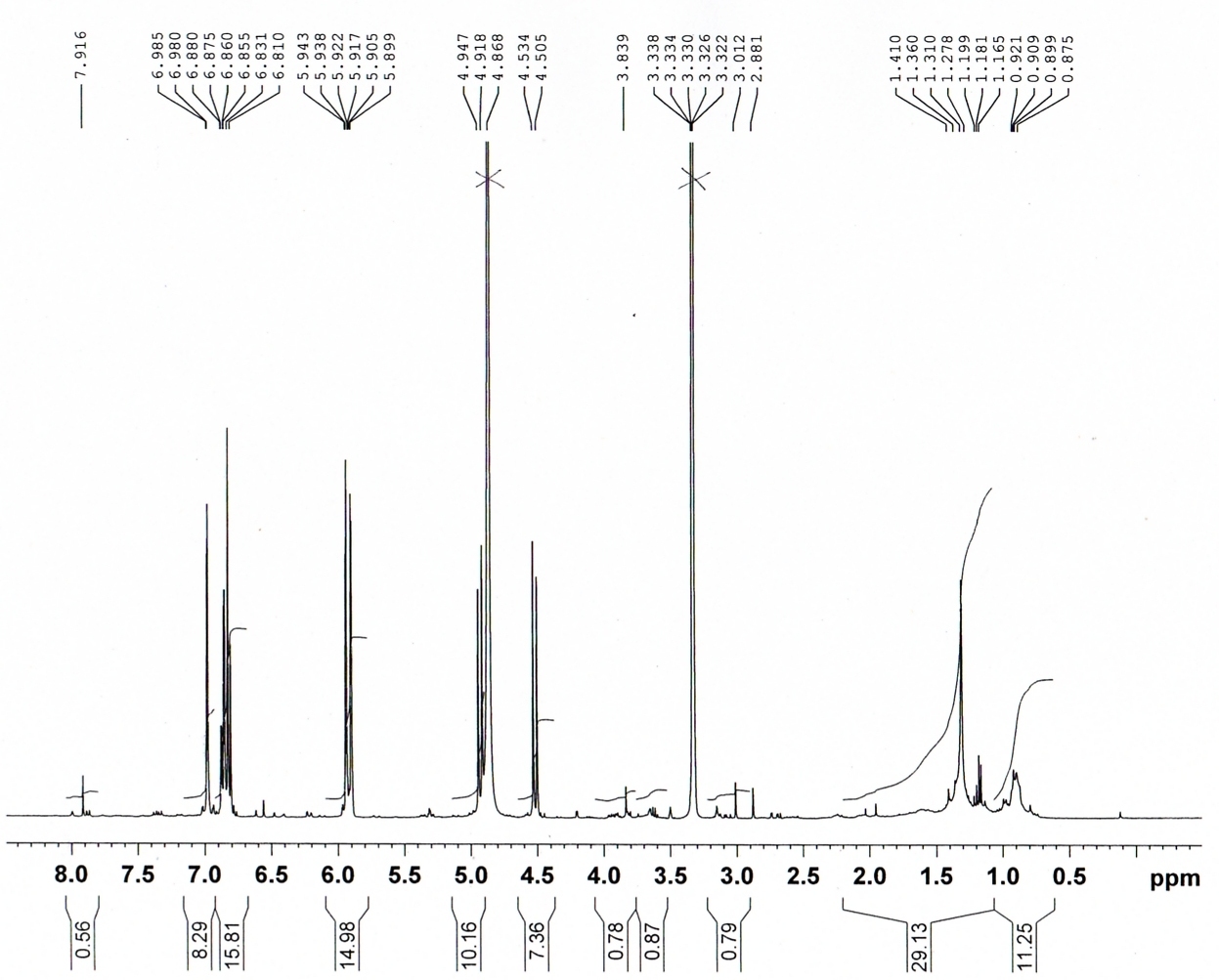


2. ^13^C-NMR spectrum (CD_3_OD-*d_4_*, 400 MHz) of (*2R*,*3R*)*-*(+)**-**taxifolin (**1**)


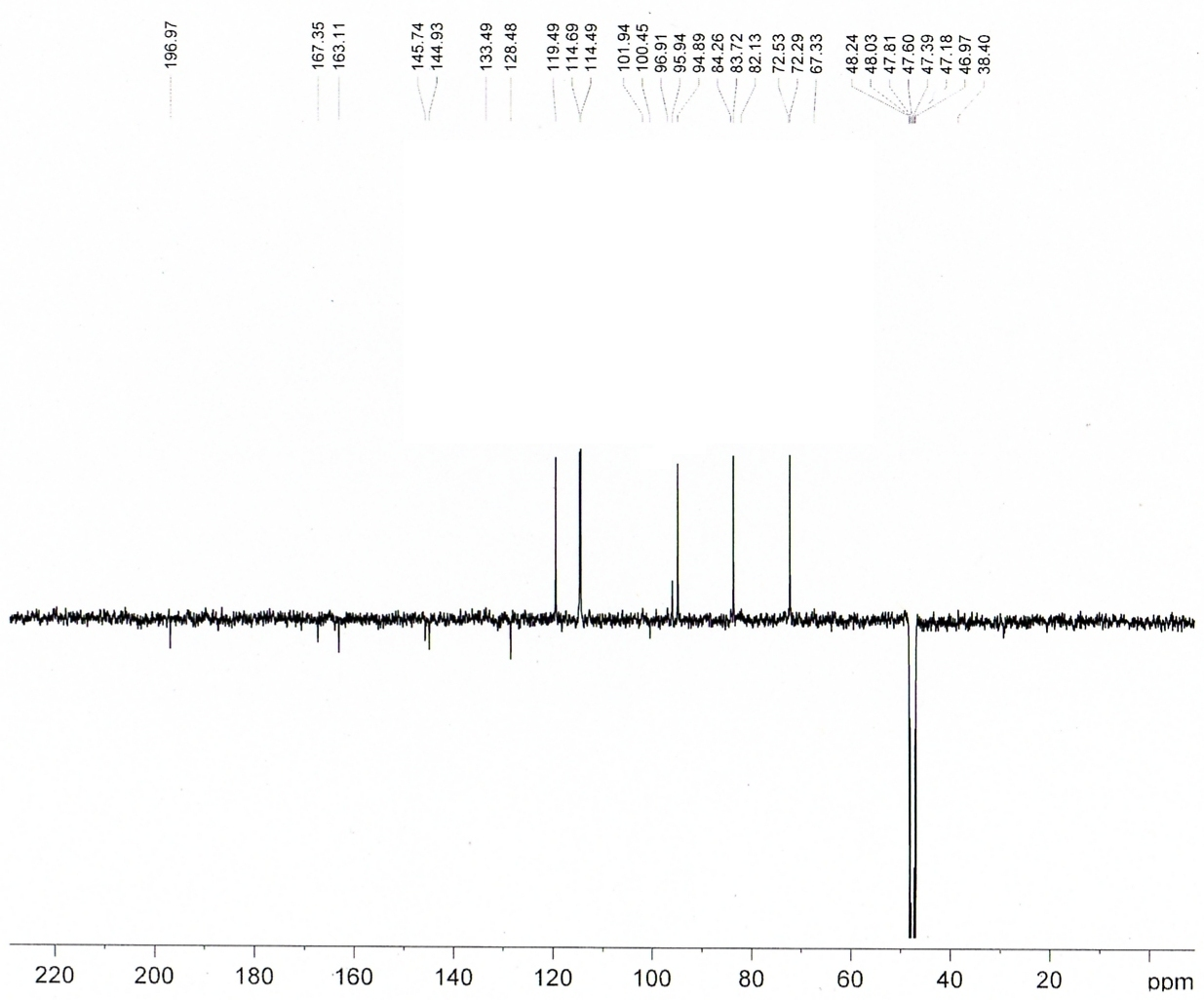


3. ^1^H-^1^H-COSY spectrum (CD_3_OD-*d_4_*, 400 MHz) of (*2R*,*3R*)*-*(+)**-**taxifolin (**1**)


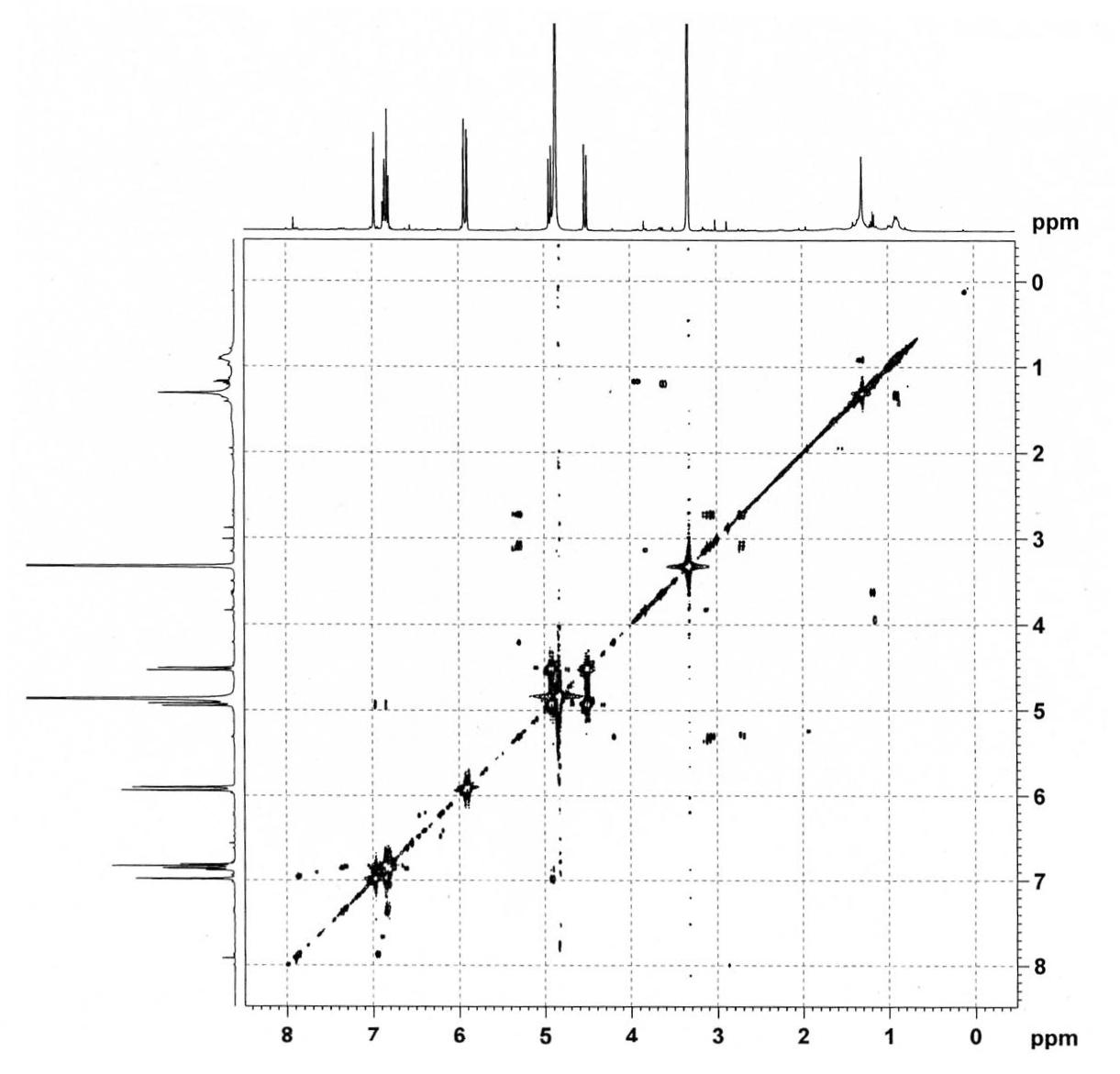


4. ^1^H-^13^C HSQC spectrum (CD_3_OD-*d_4_*, 400 MHz) of (*2R*,*3R*)*-*(+)**-**taxifolin (**1**)


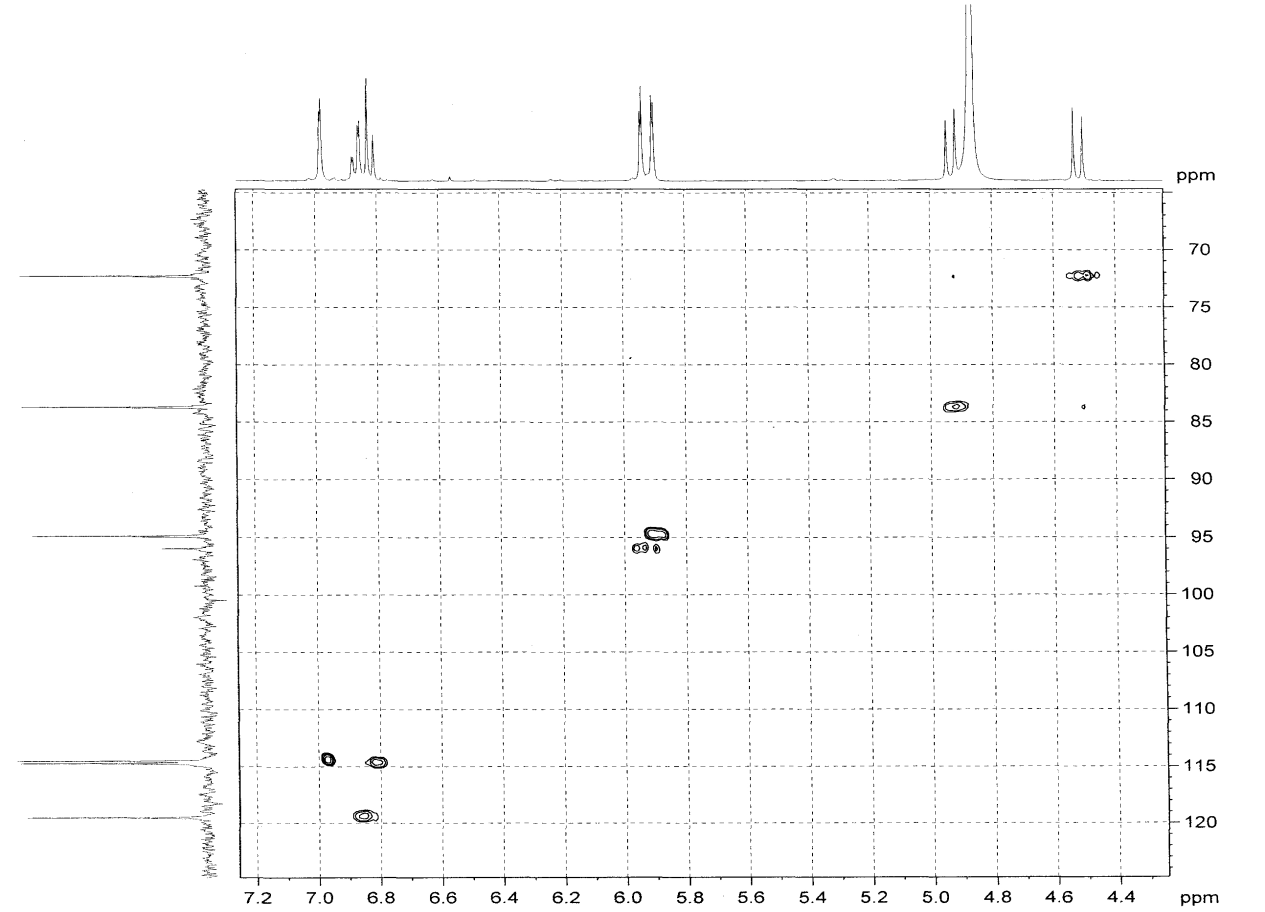


5. ^1^H-NMR spectrum (CD_3_OD-*d_4_*, 400 MHz) of (*2R*,*3R*)**-**(+)*-*aromadendrin (**2**)


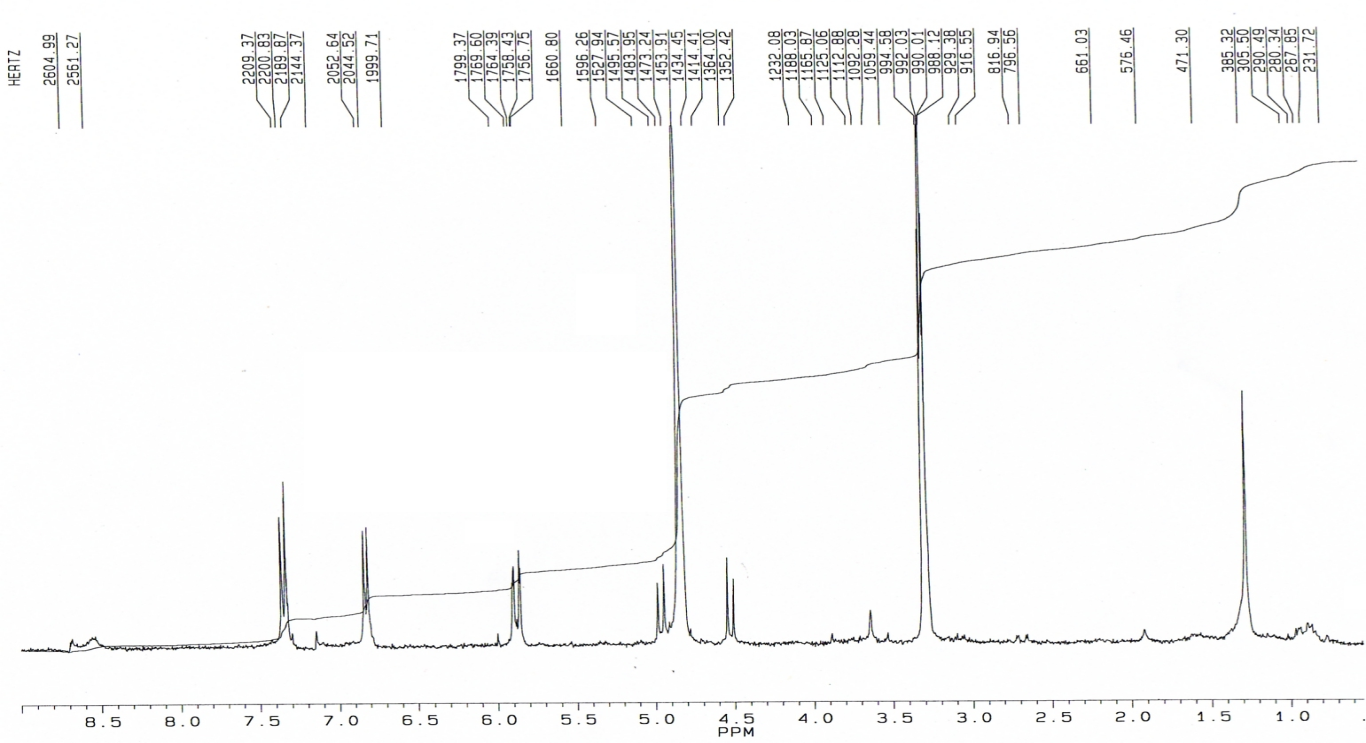


6.^1^H-NMR spectrum (CD_3_OD-*d_4_*, 400 MHz) of (*S*)-(−)-5,7,3',5'-tetrahydroxyflavanone (**3**)


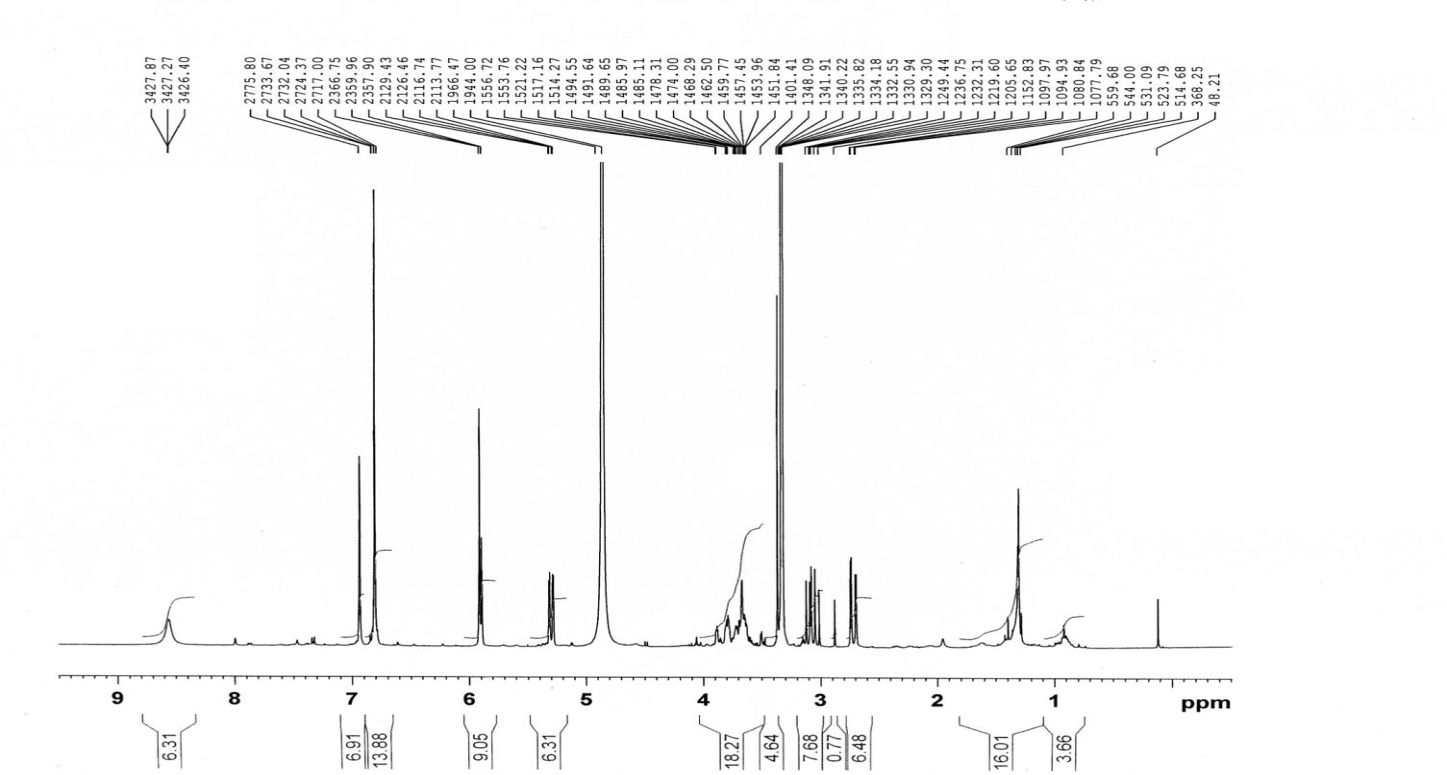


7. ^13^C-NMR (CD_3_OD-*d_4_*, 400 MHz) of (*S*)-(−)-5,7,3',5'-tetrahydroxyflavanone (**3**)


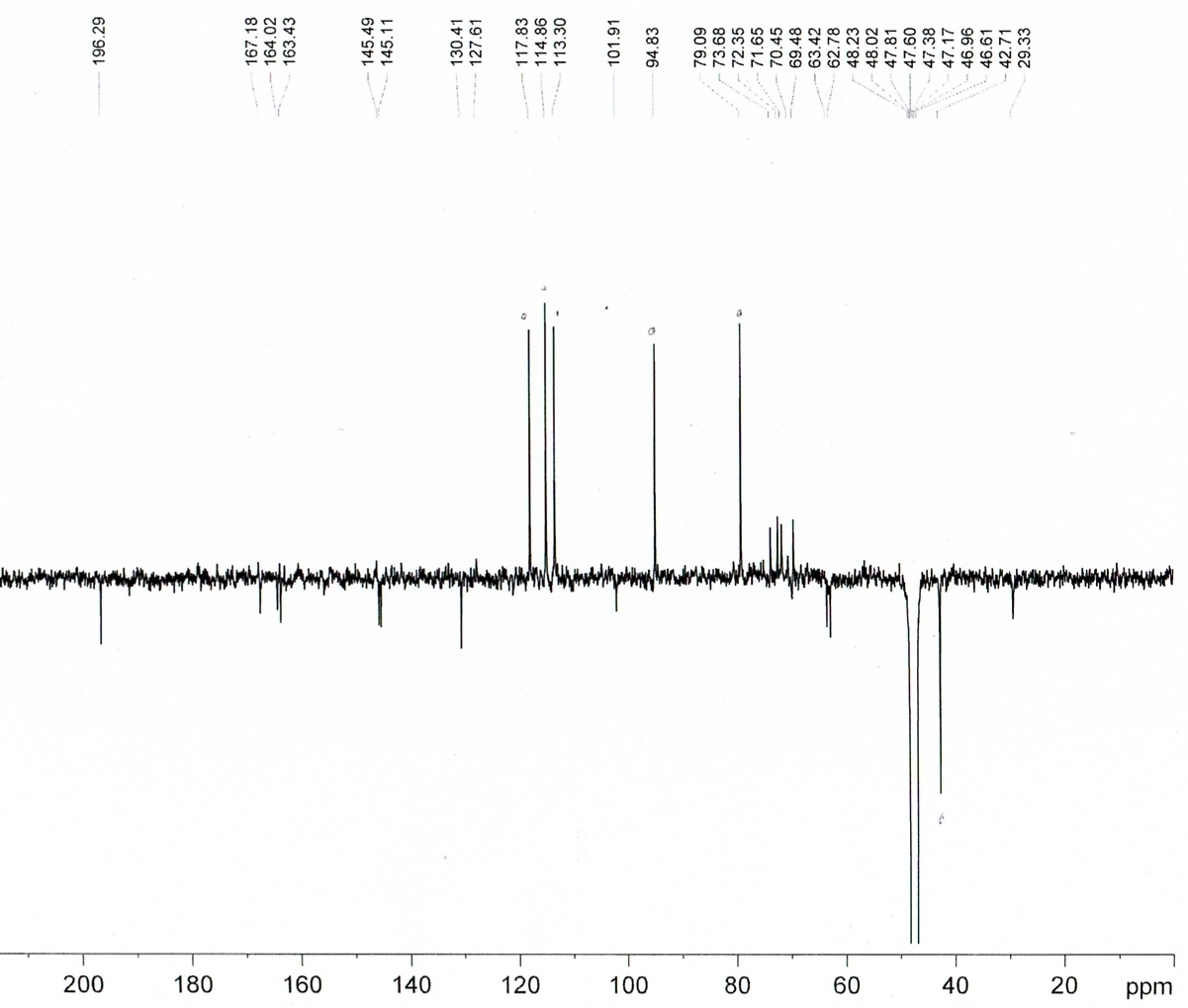


8. ^1^H-^1^H-COSY (CD_3_OD-*d_4_*, 400 MHz) of (*S*)-(−)-5,7,3',5'-tetrahydroxyflavanone (**3**)


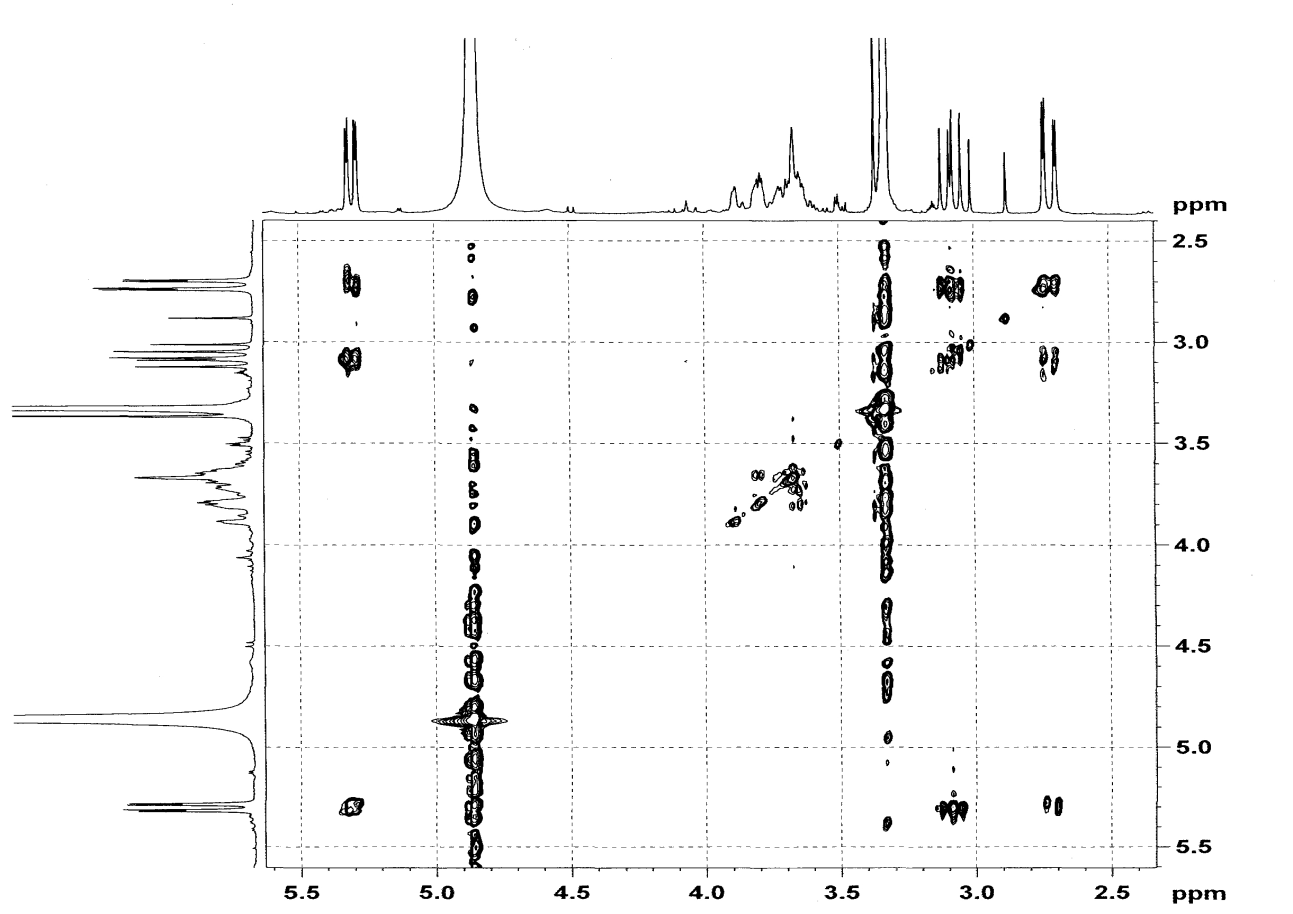


9. ^1^H-^13^C HSQC (CD_3_OD-*d_4_*, 400 MHz) of (*S*)-(−)-5,7,3',5'-tetrahydroxyflavanone (**3**)


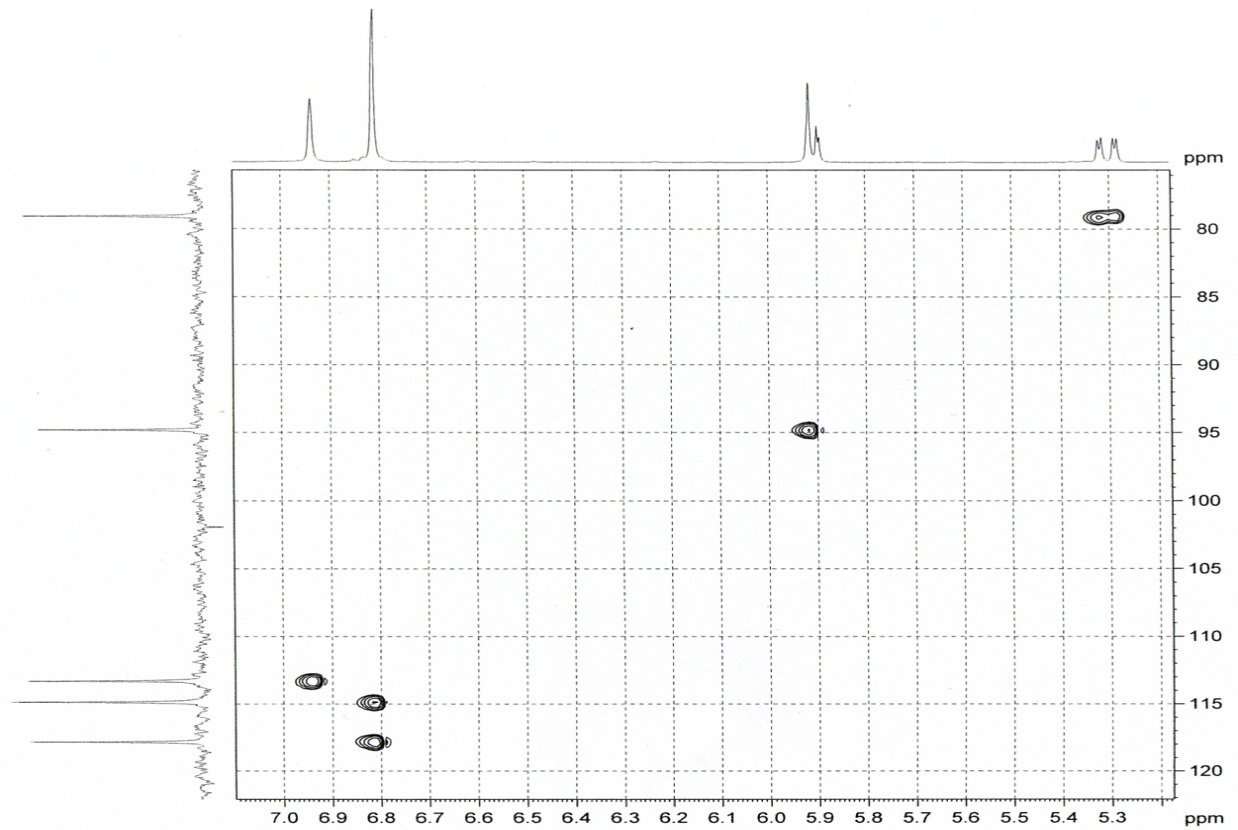


10. ^1^H-NMR spectrum (DMSO-*d_6_*, 400 MHz) of (*S*)*-*(−)**-**naringenin (**4**)

**
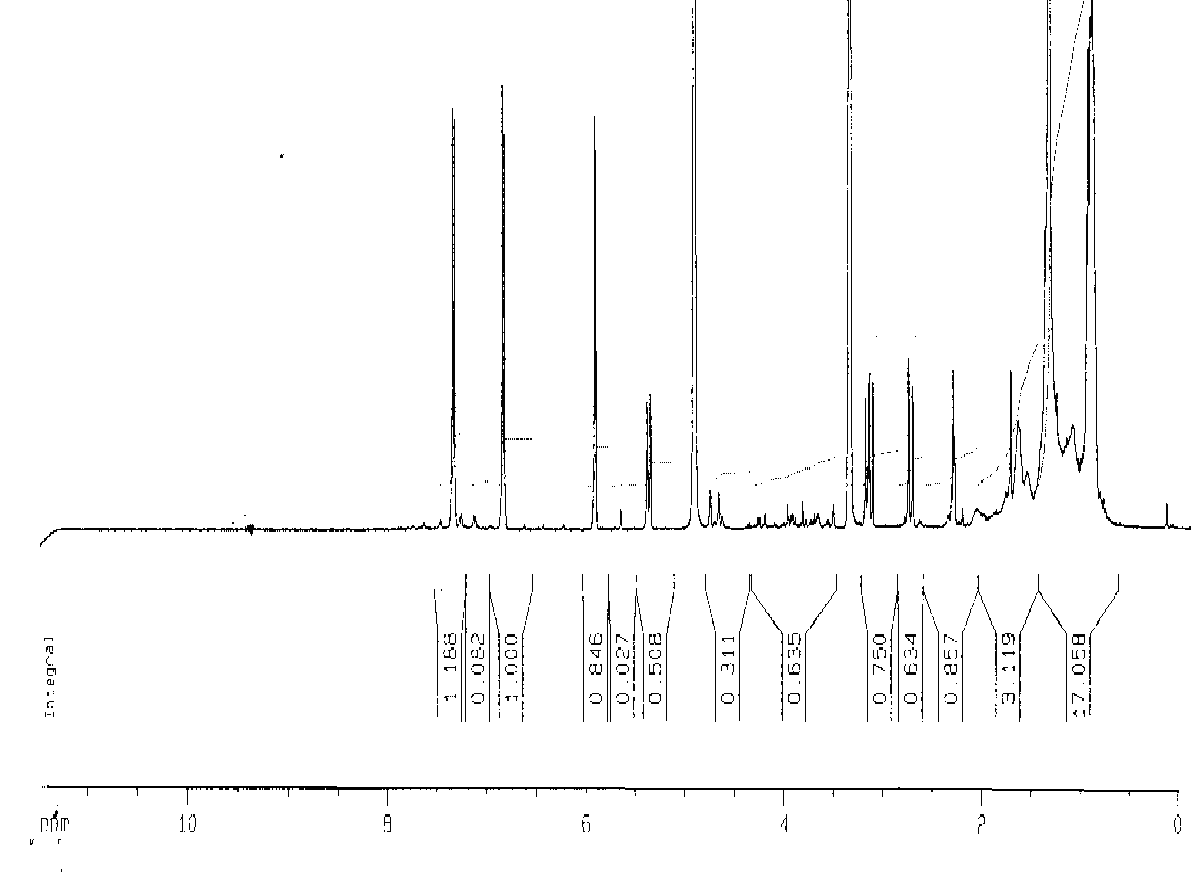
**

11. ^13^C-NMR spectrum (DMSO-*d_6_*, 400 MHz) of (*S*)*-*(−)**-**naringenin (**4**)

**
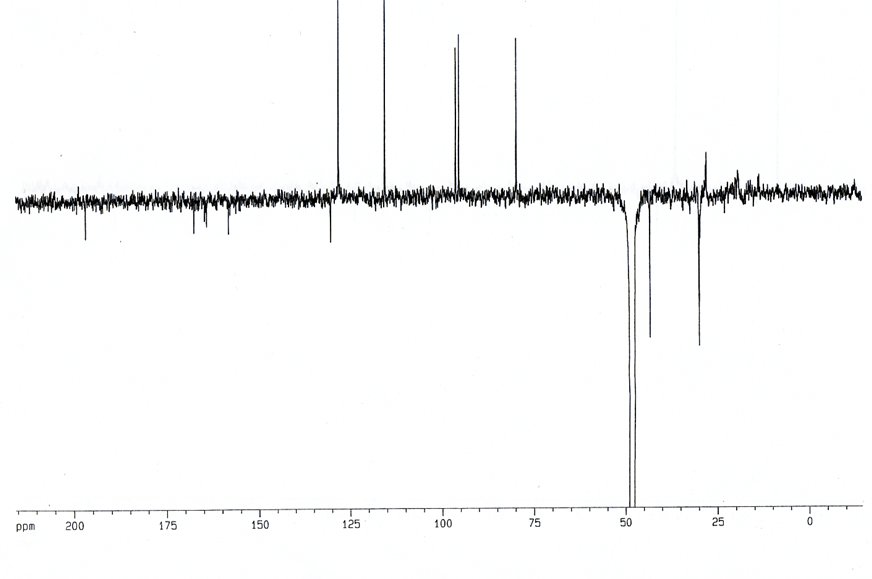
**

12. ^1^H-^1^H-COSY spectrum (DMSO-*d_6_*, 400 MHz) of (*S*)*-*(−)**-**naringenin (**4**)


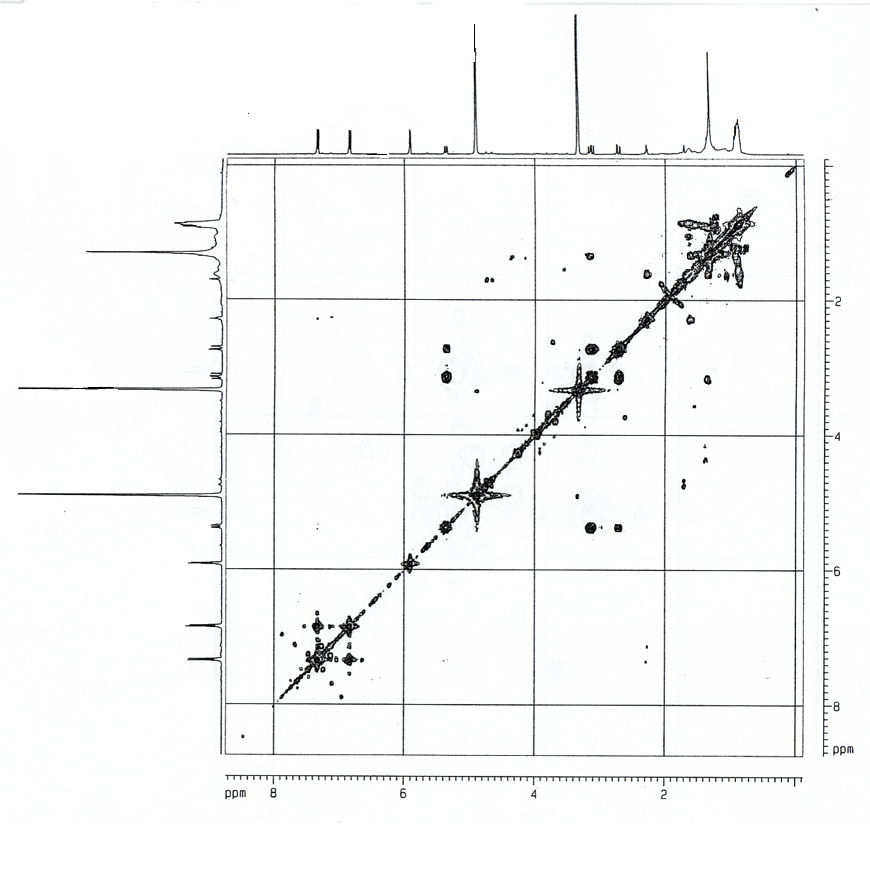


13. ^1^H-^13^C HSQC spectrum (DMSO-*d_6_*, 400 MHz) of (*S*)*-*(−)**-**naringenin (**4**)


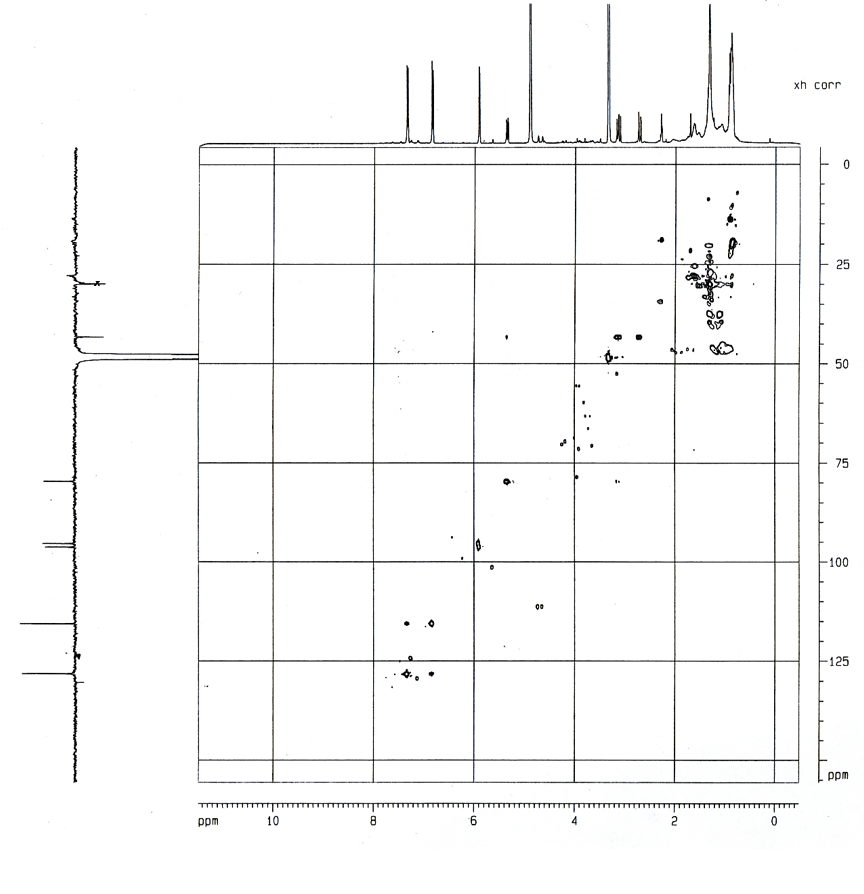

Supplement: Supplementary file 1 [file molecules-17-01665-s001.doc]
